# Inverse stochastic resonance in networks of spiking neurons

**DOI:** 10.1371/journal.pcbi.1005646

**Published:** 2017-07-10

**Authors:** Muhammet Uzuntarla, Ernest Barreto, Joaquin J. Torres

**Affiliations:** 1 Department of Biomedical Engineering, Bulent Ecevit University, Engineering Faculty, Zonguldak, Turkey; 2 Department of Physics and Astronomy and The Krasnow Institute for Advanced Study, George Mason University, Fairfax, Virginia, United States of America; 3 Department of Electromagnetism and Physics of Matter, and Institute Carlos I for Theoretical and Computational Physics, University of Granada, Granada, Spain; SUNY Downstate MC, UNITED STATES

## Abstract

Inverse Stochastic Resonance (ISR) is a phenomenon in which the average spiking rate of a neuron exhibits a minimum with respect to noise. ISR has been studied in individual neurons, but here, we investigate ISR in scale-free networks, where the average spiking rate is calculated over the neuronal population. We use Hodgkin-Huxley model neurons with channel noise (i.e., stochastic gating variable dynamics), and the network connectivity is implemented via electrical or chemical connections (i.e., gap junctions or excitatory/inhibitory synapses). We find that the emergence of ISR depends on the interplay between each neuron’s intrinsic dynamical structure, channel noise, and network inputs, where the latter in turn depend on network structure parameters. We observe that with weak gap junction or excitatory synaptic coupling, network heterogeneity and sparseness tend to favor the emergence of ISR. With inhibitory coupling, ISR is quite robust. We also identify dynamical mechanisms that underlie various features of this ISR behavior. Our results suggest possible ways of experimentally observing ISR in actual neuronal systems.

## Author summary

The rhythmic activity of spiking neurons is known to be sensitive to noise. In such neurons, inverse stochastic resonance (ISR), in which the average spiking activity of a neuron exhibits a pronounced minimum as the noise intensity increases, can occur. But macroscopic phenomena such as information processing, cognition, and mental diseases take place at the level of populations of neurons, and, presumably are strongly influenced by the connectivity structure of the network. Here we investigate the occurrence of ISR in large populations of networked spiking neurons. We find that ISR can emerge in such networks as a consequence of many different factors, including channel noise, connection strength, synaptic currents with excitatory and inhibitory terms, and topological features of the network including degree distribution and mean connectivity degree. We describe the dynamical mechanisms that give rise to various features of ISR in such networks. We find that inhibitory coupled networks favor ISR behavior and are more robust to changes in neural circuit features than electrically or excitatory coupled networks. Our work suggests that network ISR may be observable in local field potential measurements of neuronal activity in brain slice preparations, cultures, and perhaps even in EEG recordings.

## Introduction

Noise is ubiquitous in the nervous system of living organisms, yet it remains unclear how noise influences neuronal information processing. While noise is generally considered to be something that should be filtered out or reduced, it is now widely accepted that noise can, in some cases, enrich the stochastic dynamics of neuronal ensembles and facilitate their information processing capabilities [1–3]. A well-known example of this is stochastic resonance (SR), in which a certain amount of noise can enhance the detection and transmission efficiency of weak signals [4–11]. In this scenario, for low noise levels, a system does not respond to a weak signal due to its small amplitude. For moderate noise levels, however, the noise raises the inputs closer to a threshold and thereby enhances signal detection. Finally, for high noise levels, the system’s response is dominated by the noise, and therefore the signal is not detected. Thus, a plot of the system’s response versus noise is bell-shaped, indicating that there is an optimal value of the noise for signal detection and processing.

An interesting observation was reported in [12], where noise was found to have an inhibitory effect on neuronal pacemaker activity in an *in vitro* preparation of squid axon. The authors also found that small noisy input currents could induce switching between repetitive firing and quiescent neuronal states, and that the timing of the switching depended on the intensity and spectral properties of the noise. The effects of noise on the rhythmic firing activity of a pacemaker cell were also studied theoretically in a Hodgkin-Huxley model neuron [13, 14]. These works reported that near the onset of firing, a minimum—possibly zero—occurred in the average spiking activity of the model neuron with respect to noise intensity. Since this behavior is essentially the reverse of SR, the authors called this phenomenon “inverse stochastic resonance” (ISR). Recently, in [15], the impact of the temporal structure of noise on ISR was investigated and the inhibitory effect of colored noise was found to be stronger than that of the Gaussian white noise studied in [13, 14]. Furthermore, in [16], ISR studies were extended to the case of a spatially-extended Hodgkin-Huxley system. These authors showed that if the noise and signal inputs were uniformly distributed along the spatial extent of the neuron, weak noise could strongly inhibit the occurrence of rhythmic spiking, but not its propagation. However, if the noise and signal inputs were applied to separate regions of the neuron, the noise had no effect on either rhythmic spiking or the propagation of spikes. Other authors considered the Morris-Lecar model neuron [17], and showed that ISR can emerge as a consequence of unreliable spike transmission [18]. In another work [19], the authors studied the phenomenon of ISR in the Hodgkin-Huxley (HH) neuron based on biophysically realistic ion channel noise. These authors also clarified the dynamical structure that underlies ISR. More recently, a double inverse stochastic resonance (DISR) was reported in the response of a HH neuron that receives synaptic inputs subject to different types of short-term synaptic plasticity [20].

Although the ISR phenomenon has been studied extensively at the level of a single neuron, to our knowledge there is no record investigating ISR at the level of large populations—only a simple system consisting of two coupled neurons has been considered to date, and features similar to single-cell ISR were reported [21]. In the current work, we are interested in whether ISR can emerge, or not, in a neuronal medium consisting of a large number of interconnected neurons with non-trivial topology. As in other biological systems [22], one might expect that the effects of voltage or ionic current fluctuations decrease when neurons are connected in such networks, as the fluctuations can spread throughout the system via electrical connections, and quickly damp out. On the other hand, for strong coupling, the system may become a “supercell”, and effectively behave as a single unit with a very large membrane area consisting of the entire population of ionic channels in the system. In this case, the collective dynamics would be more deterministic, and ISR could be impeded. Similarly, when neurons are coupled with electrical connections, they become more synchronized, which also decreases the effect of noise in the coupled system [23]. On the the other hand, imperfect synchronization of afferents onto a postsynaptic neuron (due for instance to spike train irregularity or other sources of randomness) introduces current fluctuations whose relative amplitude with respect to the mean synaptic current scales as 〈*k*〉^−1/2^, where 〈*k*〉 is the mean number of presynaptic neighbors. This is another factor that can influence the emergence of ISR in networked systems. If the network is sparse, or the system is near a critical or a bifurcation point (as is the case in the ISR phenomenon), these network fluctuations are not negligible and can be very important in determining the appearance and features of ISR.

Therefore, whether or not ISR can emerge in networked neuronal media is an interesting issue to investigate, especially due to the possible effects that ISR might have in the context of information coding and processing. The work presented here constitutes a first step towards investigating the main factors and dynamical processes involved in ISR emergence in a large neuronal population. This work can be easily extended to explore how other factors present in a neuronal medium, such as other network topologies, degree correlations, different types of synaptic dynamics and plasticity, etc., influence ISR.

## Models and methods

The time evolution of the transmembrane potential of the HH neurons in our network is given by:

$$\begin{array}{c} C_{m}\frac{dV_{i}}{dt}=-G_{i}^{Na}\left(m_{i},\;h_{i}\right)(V_{i}-E_{Na})-G_{i}^{K}\left(n_{i}\right)(V_{i}-E_{K})-G_{i}^{L}(V_{i}-E_{L})+I_{0}+I_{i}^{syn} \end{array}$$ (1)

where *V*_*i*_ denotes the membrane potential of *i*-th neuron in millivolts, *i* = 1, …, *N*, and *C*_*m*_ = 1*μF*/*cm*^2^ is the membrane capacitance per unit area. *I*_0_, measured in *μA*/*cm*^2^, is an external bias current injected into all neurons in the network and is used for the modulation of neuronal excitability. In our study, we set *I*_0_ = 6.8 *μA*/*cm*^2^, for which the neuron exhibits bistability between a resting and a spiking state. The parameters *E*_*Na*_ = 115*mV*, *E*_*K*_ = −12*mV*, and *E*_*L*_ = 10.6*mV* are the reversal potentials for sodium, potassium and leak channels, respectively. $G_{i}^{Na}$, $G_{i}^{K}$, and $G_{i}^{L}$ represent the corresponding channel conductances. In the model, the leak conductance is assumed to be constant, with $G_{i}^{L}=0.3\;mS/cm^{2}$, while the sodium and potassium conductances vary according to the following equations [24]:

$$\begin{array}{c} G_{i}^{Na}\left(m,\;h\right)=g_{Na}^{max}m_{i}^{3}h_{i} \end{array}$$ (2)

$$\begin{array}{c} G_{i}^{K}\left(n\right)=g_{K}^{max}n_{i}^{4}. \end{array}$$ (3)

Here, $g_{Na}^{max}=120\;mS/cm^{2}$ and $g_{K}^{max}=36\;mS/cm^{2}$ are the maximal sodium and potassium conductances. *m*_*i*_ and *h*_*i*_ are the activation and inactivation gating variables of the sodium channel, respectively, and the product $m_{i}^{3}h_{i}$ represents the mean proportion of open sodium channels in the membrane patch of neuron *i*. The potassium channel includes an activation gating variable *n*_*i*_, and similarly, $n_{i}^{4}$ is the mean proportion of open potassium channels in neuron *i*.

To incorporate ion channel noise into the dynamics of each individual neuron, we use the well-known algorithm proposed by Fox [25], both because it is widely used (e.g., [18, 26–29]), and because it is computationally efficient. Since we consider a large range of model parameters (patch size, synaptic coupling, network parameters) throughout this work, the latter consideration is important. The approach put forward by [25] expresses the gating variable dynamics in terms of Langevin equations as follows:

$$\begin{array}{c} dx_{i}/dt=\alpha_{x_{i}}\left(1-x_{i}\right)-\beta_{x_{i}}x_{i}+\xi_{x_{i}}\left(t\right), \end{array}$$ (4)

where $\alpha_{x_{i}}$ and $\beta_{x_{i}}$ are rate functions for the gating variable *x*_*i*_ (*x* = *m*,*n*,*h*) and can be found in [30]. The probabilistic nature of the channels appears as a noise source $\xi_{x_{i}}(t)$ in Eq (4). This is an independent zero-mean Gaussian white noise source whose autocorrelation function is given as follows [25]:

$$\begin{array}{c} \langle \xi_{m_{i}}\left(t\right)\xi_{m_{i}}\left(t^{\prime }\right)\rangle =\left\{2\alpha_{m_{i}}\beta_{m_{i}}/\left[N_{Na}\left(\alpha_{m_{i}}+\beta_{m_{i}}\right)\right]\right\}\delta \left(t-t^{\prime }\right) \end{array}$$ (5)

$$\begin{array}{c} \langle \xi_{h_{i}}\left(t\right)\xi_{h_{i}}\left(t^{\prime }\right)\rangle =\left\{2\alpha_{h_{i}}\beta_{h_{i}}/\left[N_{Na}\left(\alpha_{h_{i}}+\beta_{h_{i}}\right)\right]\right\}\delta \left(t-t^{\prime }\right) \end{array}$$ (6)

$$\begin{array}{c} \langle \xi_{n_{i}}\left(t\right)\xi_{n_{i}}\left(t^{\prime }\right)\rangle =\left\{2\alpha_{n_{i}}\beta_{n_{i}}/\left[N_{K}\left(\alpha_{n_{i}}+\beta_{n_{i}}\right)\right]\right\}\delta \left(t-t^{\prime }\right), \end{array}$$ (7)

where *N*_*Na*_ and *N*_*K*_ represent total number of sodium and potassium channels within a membrane patch. Assuming homogeneous sodium and potassium ion channel densities of *ρ*_*Na*_ = 60*μm*^−2^ and *ρ*_*K*_ = 18*μm*^−2^ [24], the total channel numbers are calculated by *N*_*Na*_ = *ρ*_*Na*_*A*, *N*_*K*_ = *ρ*_*K*_*A*. Here, *A* is the membrane patch area of each neuron.

Note that constant ion channel densities means that for large membrane area *A*, many ion channels are involved, and accordingly the stochastic contribution of individual ion channels becomes negligible. In this case the collective dynamics of all channels approaches the deterministic description. However, when *A* is small, membrane conductance fluctuations significantly increase, since relatively fewer channels are involved [27, 31, 32]. Thus, membrane area and effective noise amplitude are inversely related. We will use *A* as the channel noise control parameter throughout this study.

Finally, in Eq (1), $I_{i}^{syn}$ denotes the total synaptic current received by neuron *i*. We consider coupling via electrical gap junctions and chemical synapses. In the case of linear electrical coupling via gap junctions, the synaptic current is proportional to the transmembrane potential difference between neuron *i* and that of its neighbor, summed over neighbors:

$$\begin{array}{c} I_{i}^{syn}=\sum_{j\epsilon \;neigh(i)}g_{e}\left(V_{j}-V_{i}\right) \end{array}$$ (8)

where *g*_*e*_ is the conductance of the gap junction, and the sum runs over the neighbors that feed the neuron *i*. For coupling via chemical synapses, the synaptic current takes the form [33]:

$$\begin{array}{c} I_{i}^{syn}=\sum_{j\epsilon \;neigh(i)}g_{c}\alpha \left(t-t_{0}^{j}\right)\left(E_{rev}-V_{i}\right), \end{array}$$ (9)

where the alpha function *α*(*t*) describes the temporal evolution of the synaptic conductance. Here, *g*_*c*_ is the maximal conductance of the synaptic channel, and $t_{0}^{j}$ is the time at which presynaptic neuron *j* fires. In our study, we use exponential synapses such that $\alpha \left(t\right)=e^{-t/\tau_{syn}}\Theta \left(t\right)$ with *τ*_*syn*_ = 3*ms*, and Θ(*t*) is the Heaviside step function. In practice, exponential synapses are implemented as follows:

$$\begin{array}{c} I_{i}^{syn}=\sum_{j\epsilon \;neigh(i)}g_{c}s_{j}\left(E_{rev}-V_{i}\right) \end{array}$$ (10)

$$\begin{array}{c} \dot{s_{j}}=-s_{j}/\tau_{syn}+\delta \left(t-t_{0}^{j}\right), \end{array}$$ (11)

where *s*_*j*_ is the fraction of open receptor channels for neuron *j*. The parameter *E*_*rev*_ is the synaptic reversal potential, and its value determines the type of synapse. If it is larger than the resting potential (around 0 mV), e.g. *E*_*rev*_ = 70*mV*, the synapse is excitatory; if it is smaller, e.g. *E*_*rev*_ = −10*mV*, the synapse is inhibitory.

We base the connection topology of our neurons on random scale-free (SF) networks. This is a well-known and widely-used connectivity paradigm in computational studies of local microcircuits, since such connectivity has been observed in many functional brain regions via neuroimaging and electrophyisiological studies [34–37]. Graph analyses of resting-state fMRI data from experimental studies have also suggested an efficient organization of functional communication in brain networks, indicating that the human brain is not just a random network, but one with an organization optimized towards a high level of local and global efficiency. This can be modeled with a SF structure [38]. In our setup, unless otherwise noted, our SF networks are composed of *N* = 200 nodes with a neuron at each node.

To construct the connectivity matrix of the SF network, we first draw *N* random numbers *k*_*i*_, which represent the connectivity degree of each neuron, from a distribution *p*(*k*) ∼ *k*^−*γ*^ with mean connectivity 〈*k*〉 in the thermodynamic limit (*N* → ∞), and which is normalized in the interval (*k*_0_, *k*_*max*_) with $k_{0}=\langle k\rangle \frac{\left(\gamma -2\right)}{\left(\gamma -1\right)}{\left(1-N^{\frac{2-\gamma }{\gamma -1}}\right)}^{-1}$ and $k_{max}=\sqrt{\langle k\rangle N}$. (See, for instance, [39]). We then set the elements of the connectivity matrix to *ϵ*_*ij*_ = *ϵ*_*ji*_ = 1 with probability $p_{ij}=\frac{k_{i}k_{j}}{N\langle k\rangle }$, and *ϵ*_*ij*_ = 0 otherwise (configurational ensemble), until the degree of each node matches the desired values *k*_*i*_, ∀*i*.

We calculate the mean firing rate of the network for fixed values of membrane area *A* and input current *I*_0_ as follows. First, we select separate initial conditions for each neuron randomly and uniformly from within a fixed region of the four-dimensional state space (*V*, *m*, *n*, and *h*). Specifically, this region ranges from −10*mV* to 80*mV* for the trans-membrane voltage variable *V*, and from 0 to 1 for each of the gating variables *m*, *n*, and *h*. Then, the network equations are integrated for a time *T* = 1 s. After this, for each neuron, we count the number of spikes $N_{i}^{spikes}$ that occur in an additional time interval of length *τ* = 5 s. Each spiking event is defined by the upward crossing of the membrane potential past a threshold of 20 mV. The mean spiking rate is then calculated by:

$$\begin{array}{c} \nu =\frac{1}{N\tau }(\sum_{i=1}^{N}N_{i}^{spikes}), \end{array}$$ (12)

where the index *i* refers to each neuron.

To quantify the synchronization of the network, we use a dimensionless synchrony measure ***sync*** as in [40–42]. For a given time *t*, the average membrane potential in the network can be evaluated by the following equation:

$$\begin{array}{c} \bar{V}\left(t\right)\equiv \frac{1}{N}\sum_{i=1}^{N}V_{i}\left(t\right) \end{array}$$ (13)

and its time fluctuation can be characterized by the variance

$$\begin{array}{c} \Delta ={\langle \bar{V}{\left(t\right)}^{2}\rangle }_{t}-{\langle \bar{V}\left(t\right)\rangle }_{t}^{2} \end{array}$$ (14)

where 〈⋅〉_*t*_ denotes averaging over time. To normalize this quantity, we calculate the variance of the membrane voltage of a single neuron,

$$\begin{array}{c} \Delta_{i}={\langle V_{i}{\left(t\right)}^{2}\rangle }_{t}-{\langle V_{i}\left(t\right)\rangle }_{t}^{2} \end{array}$$ (15)

and construct the synchronization measure

$$\begin{array}{c} sync=\frac{\Delta }{\frac{1}{N}\sum_{i=1}^{N}\Delta_{i}} \end{array}$$ (16)

This quantity is a dimensionless value between 0 and 1 and measures the degree of coherence in the system in the infinite size limit (*N* → ∞), with larger values indicating more synchronization in the network.

The model is integrated numerically using the fourth-order Runge-Kutta algorithm with a fixed temporal resolution of 10 *μs*. For statistical accuracy, each data point in the following results is obtained by averaging 50 independent network realizations.

## Results

### ISR in networks with electrical synapses

**Fig 1:**
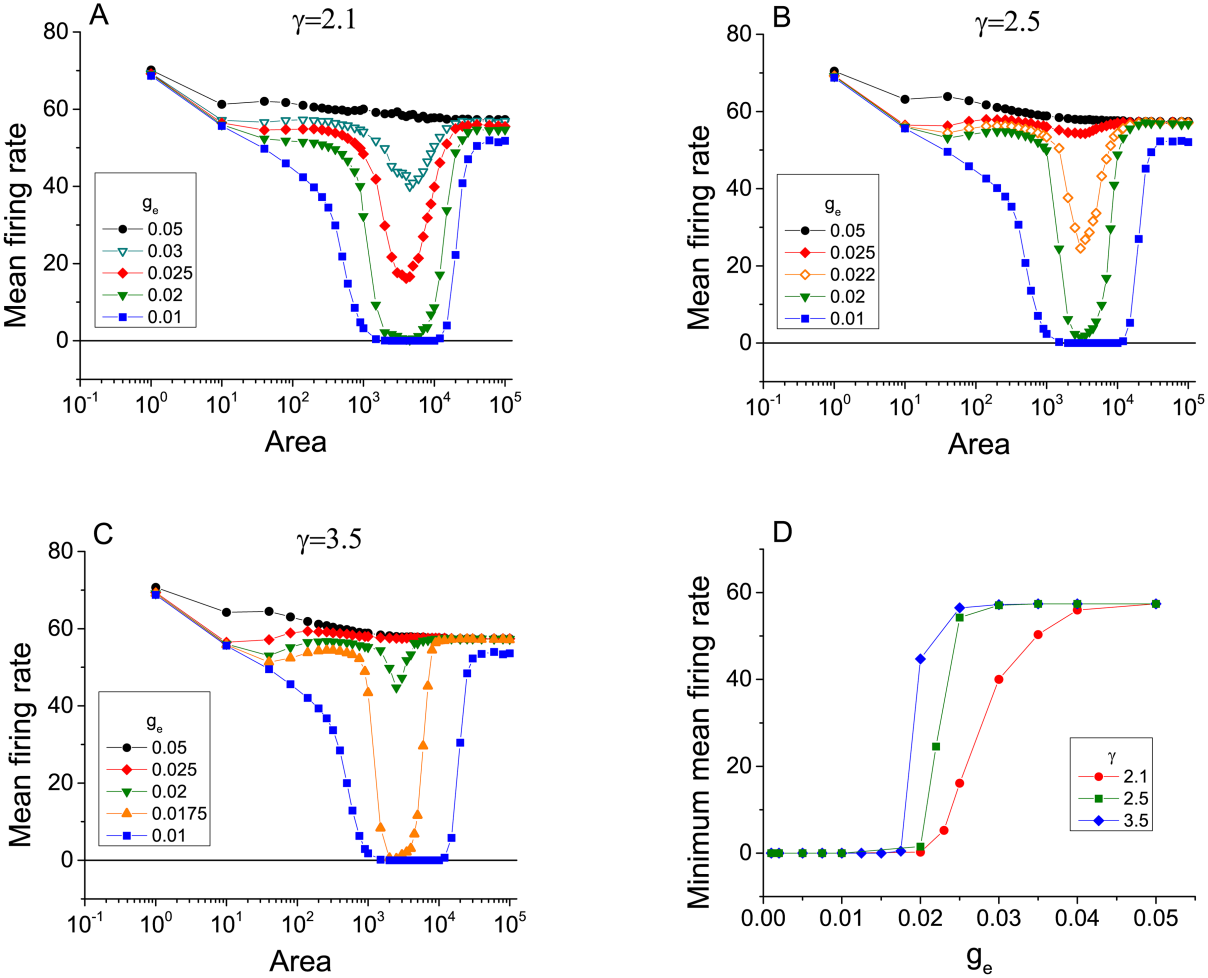
The emergence of ISR in scale-free networks of electrically-coupled neurons for different levels of network heterogenity. The panels show the mean firing rate as a function of cell membrane area for various values of gap junction conductance *g*_*e*_, with networks having degree distribution *p*(*k*) ∼ *k*^−*γ*^ and mean connectivity degree 〈*k*〉 = 5. Analysis has been performed using three different values of degree distribution exponent *γ*, (A) *γ* = 2.1, (B) *γ* = 2.5, and (C) *γ* = 3.5. Note that lower values of *γ* indicates a more heterogenous network in terms of connectivity. (D) The minimum values of the mean firing rates in panels (A)-(C) versus the gap junction conductance *g*_*e*_. As *γ* increases, the transition becomes sharper and occurs at lower values of *g*_*e*_.

**Fig 2:**
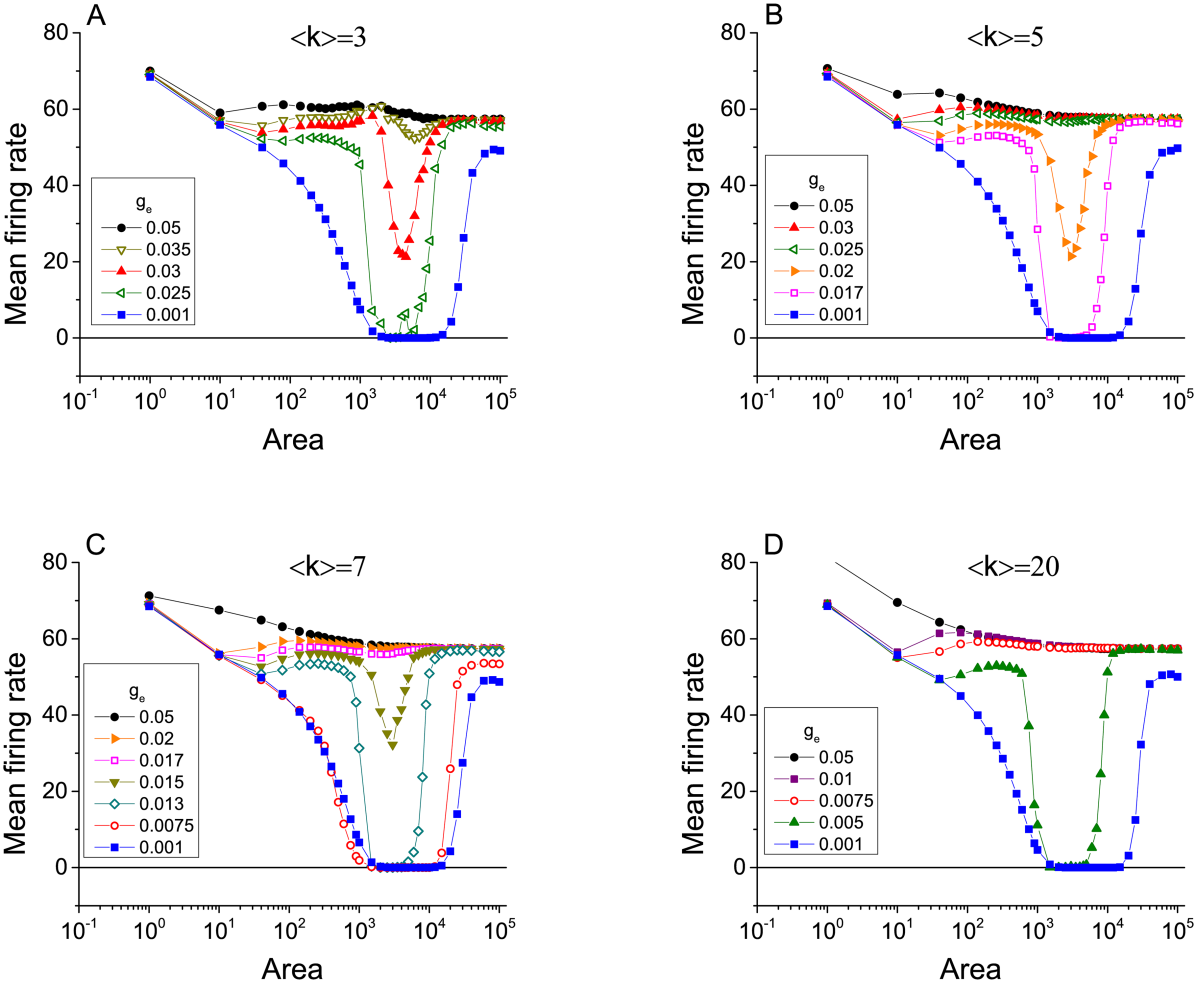
The emergence of ISR in scale-free networks of electrically-coupled neurons for different levels of mean connectivity. The panels show the mean firing rate as a function of cell membrane area for various values of gap junction conductance *g*_*e*_, with networks having degree distribution *p*(*k*) ∼ *k*^−*γ*^ and mean connectivity degree 〈*k*〉. Analysis has been performed in networks with fixed *γ* = 3, and four different values of 〈*k*〉, (A) 〈*k*〉 = 3, (B) 〈*k*〉 = 5, (C) 〈*k*〉 = 7, and (D) 〈*k*〉 = 20.

We begin by exploring the emergence of ISR in scale-free networks coupled via gap junctions as in Eq 8. We find that indeed, ISR appears for a range of gap junction conductances and network topology parameters, as shown in Figs 1 and 2. In both figures, the mean firing rate of the neurons in the network is plotted versus membrane area for various values of the gap junction conductance *g*_*e*_ (Note that the horizontal axis can be interpreted as the noise level, with noise decreasing as membrane area increases.). Fig 1 considers networks with mean connectivity degree 〈*k*〉 = 5 and various values of the degree distribution exponent *γ* (panels A-C). Fig 2 considers networks with *γ* = 3 and various values of 〈*k*〉. In all cases, the ISR effect is seen most prominently for low values of *g*_*e*_. In particular, for low *g*_*e*_ we observe that for increasing *A* (that is, decreasing noise), the mean firing rate first decreases, reaches a minimum, and then increases again. This is the ISR phenomenon. For the lowest values of *g*_*e*_, the mean firing rate remains very low, and in some cases is actually zero, over a significant interval of *A* (i.e., channel noise amplitude).

**Fig 3:**
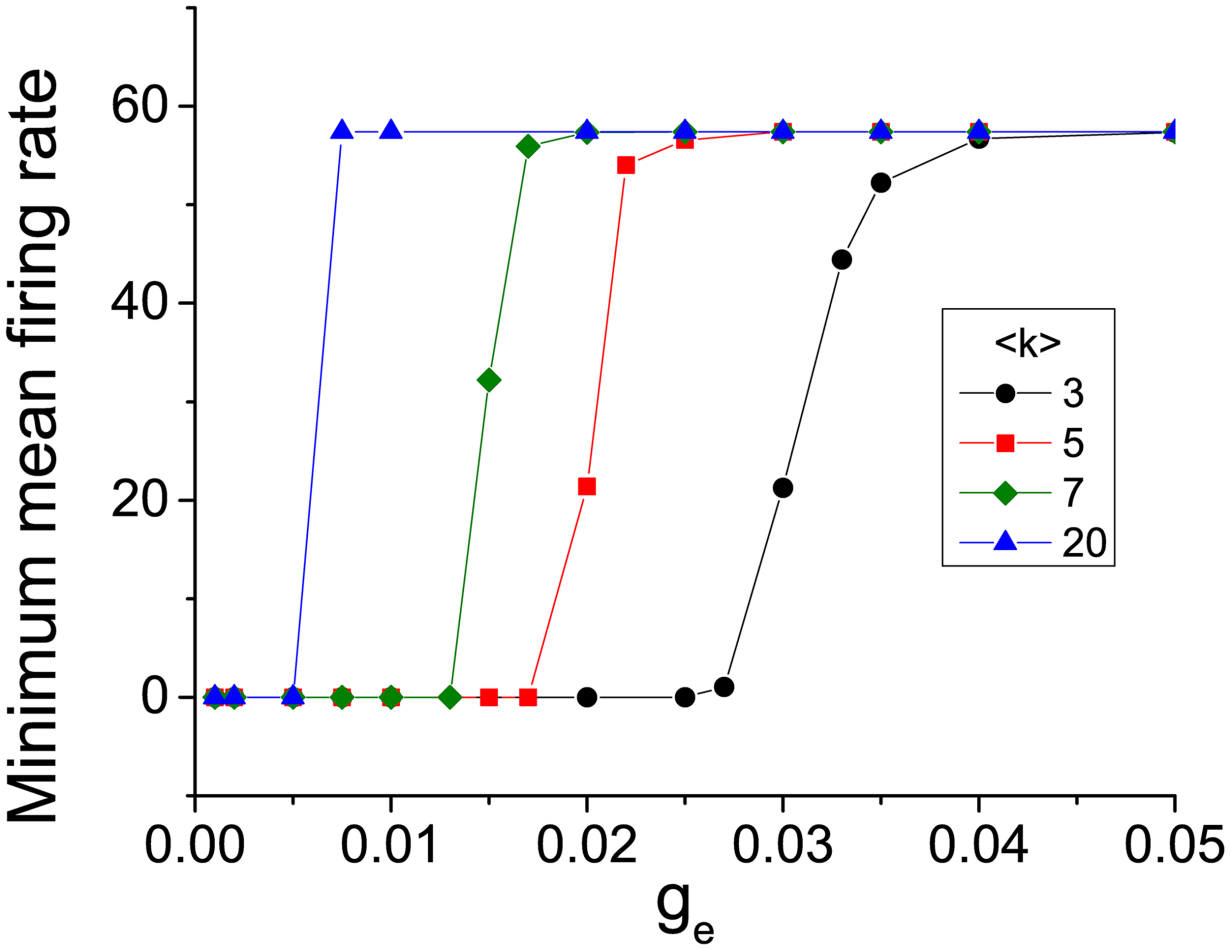
Transition from the appearance of ISR to its disappearance in networks of electrically coupled neurons with different values of mean connectivity. The plots depict the minimum values of the mean spiking rate in panels (A)-(D) of Fig 2 versus the gap junction conductance *g*_*e*_. As 〈*k*〉 increases, the transition becomes sharper and occurs at lower values of *g*_*e*_.

We observe that as *g*_*e*_ increases, the ISR effect gradually disappears in the sense that the dip in the mean firing rate is less and less pronounced. Indeed, for the largest values of *g*_*e*_ that we investigated, ISR is not apparent at all. This observation is summarized in Figs 1D and 3, which show the minimum values of the mean firing rate over the entire range of *A* studied versus the gap junction conductance. As *g*_*e*_ increases from zero, this minimum firing rate remains very low, and then quickly increases. Note that for low values of *γ* and *k*, the transition is more gradual. The transition becomes sharper for larger values of these parameters. Furthermore, the critical value of *g*_*e*_ for this transition decreases as these same parameters increase. This last observation is most evident in Fig 3.

Lower values of the degree distribution exponent *γ* correspond to a wider range of possible node degrees, indicating more heterogeneous networks in terms of connectivity. We conclude then that more heterogeneous networks favor the occurrence of ISR. By this we mean that it occurs over a wider range of *g*_*e*_, as compared to more homogenous and strongly coupled networks. Fig 1D shows that for 〈*k*〉 = 5 and *γ* = 2.1, ISR can be observed for values of *g*_*e*_ ∈ [0, 0.04], approximately, whereas for *γ* = 3.5, ISR only occurs in an interval half as big, i.e., for *g*_*e*_ ∈ [0, 0.02]. On the other hand, we can also conclude by inspecting Fig 3 that for a certain level of heterogeneity in the network, sparser connectivity favors the emergence of ISR for a wide range of *g*_*e*_. For instance, Fig 3 shows that ISR can be observed for *g*_*e*_ ∈ [0, 0.04] for 〈*k*〉 = 3 whereas for 〈*k*〉 = 20, ISR occurs only in *g*_*e*_ ∈ [0, 0.005].

**Fig 4:**
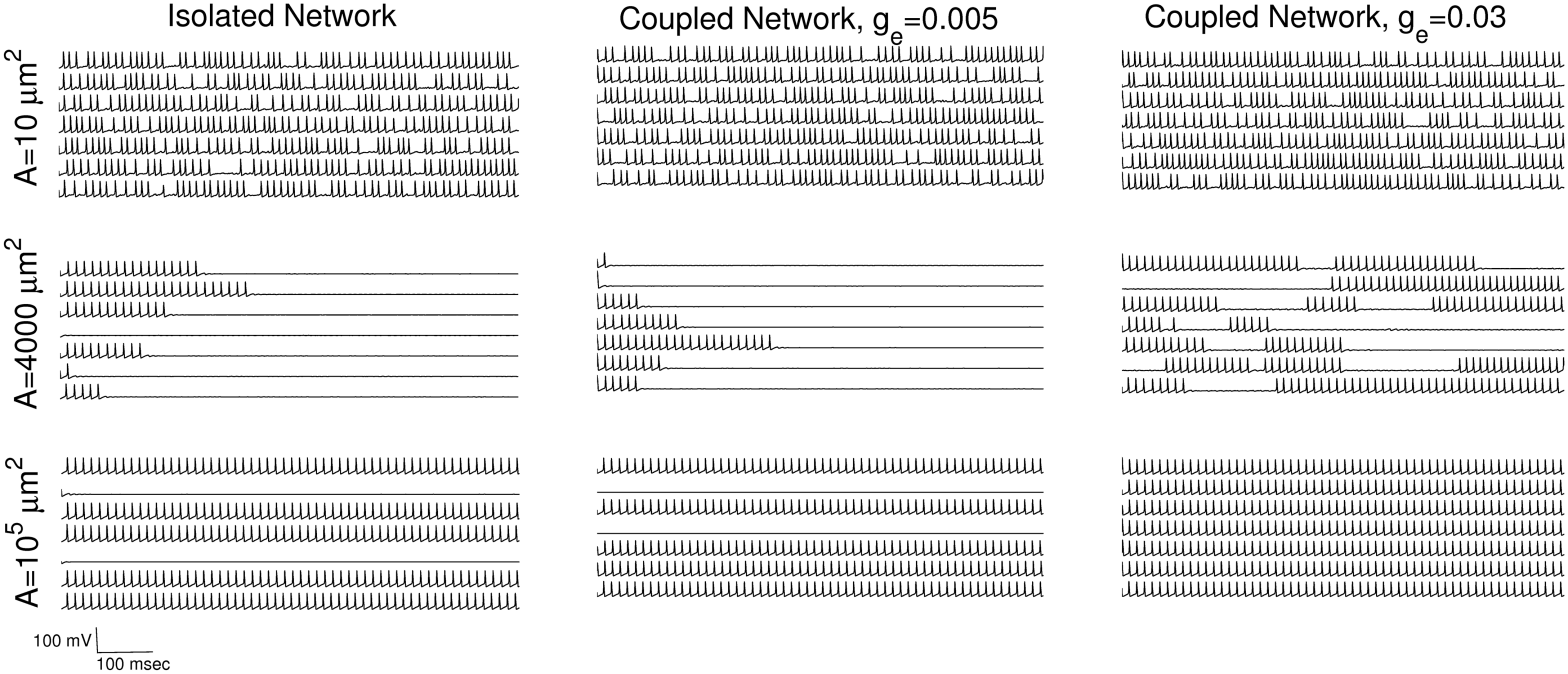
Behavior of the neurons in the network for different gap junction strengths and internal noise levels. Spike trains from seven randomly-selected neurons in a network with parameters 〈*k*〉 = 3 and *γ* = 3, for various values of *A* (rows) and *g*_*e*_ (columns) as shown. Reading downward, the columns show spiking patterns as the channel noise decreases. The left column has *g*_*e*_ = 0, and therefore the network consists of isolated neurons. The middle and right columns show behaviors with weak coupling (*g*_*e*_ = 0.005) and relatively larger coupling (*g*_*e*_ = 0.03), respectively.

To gain an understanding of the basic mechanisms behind the occurrence of ISR, and in particular its disappearance in networks with increasing gap junction conductance (as shown above), we examined the behavior of seven randomly-chosen neurons from a network with 〈*k*〉 = 3 and *γ* = 3 (i.e, the case depicted in Fig 2A). Voltage traces for these neurons at different values of *g*_*e*_ and *A* are shown in Fig 4. The left column shows the case for *g*_*e*_ = 0, corresponding to a network of isolated neurons. The middle column has *g*_*e*_ = 0.005, corresponding to a network in which the ISR effect is quite pronounced, and the right column has *g*_*e*_ = 0.03, for which the ISR effect is less pronounced.

The occurrence of ISR in a network of isolated neurons (i.e., with *g*_*e*_ = 0; see left column in Fig 4) is equivalent to ISR in a single neuron, in which the mean firing rate is obtained by averaging over repeated trials (with different initial conditions). The fundamental mechanisms at play in this case were described in detail in [19], and we briefly summarize the main points here. In the case of small membrane area, i.e., large noise, the neurons in the network spike at a high rate, but randomly, with essentially no quiescent periods. This can be seen in the top traces of the left column of Fig 4. We observed that as the noise decreases (or *A* increases), brief, random periods of quiescence appear, and these get increasingly longer in duration as the noise level decreases (similar to the middle panel, right column of Fig 4). Eventually these periods of quiescence become effectively permanent, as shown in the middle traces, left column of Fig 4. The noise levels at which this occurs correspond to the lowest portions of the ISR curve, i.e., the lowest values of mean firing rate.

The effective permanence of these quiescent periods can be understood as follows. The neurons exhibit bistability at the chosen parameters (here, *I*_0_ = 6.8 *μA*/*cm*^2^) such that a stable equilibrium (corresponding to the resting state) coexists with a stable limit cycle (corresponding to the spiking state). These are separated by a boundary, which is mediated by an unstable limit cycle. (For a more detailed description of the HH model bifurcation diagram, see for instance [19, 20]). Notably, the minimum distance from the stable equilibrium to this boundary is much larger than the minimum distance between the boundary and the stable limit cycle. Thus, for low noise levels, a neuron in the spiking state is likely to encounter a noise kick that sends it across the boundary and into the basin of the stable equilibrium, where it becomes trapped. Once trapped, this low-amplitude noise is not large enough to kick the neuron back into the spiking state with any reasonable probability. Thus, after a sufficient amount of time, the majority of neurons in the isolated network under these conditions become quiescent, and hence the mean firing rate becomes small, if not zero. We call this mechanism the **“trapping effect”**.

As the noise amplitude continues to decrease, the mean firing rate grows due to a mechanism that we call the **“initial condition effect”**. With very small noise, neurons which are initiated in a spiking state remain spiking, and neurons that begin at rest remain at rest. This can be seen in the lower traces, left column, of Fig 4. Thus, the mean firing rate calculated over all the neurons of the isolated network increases as the noise amplitude goes to zero (i.e., large membrane area), eventually saturating at a value related to the proportion of initial conditions that are in the basin of the spiking state [19].

On the other hand, neurons in networks with non-zero coupling experience two sources of noise: the channel noise, here parameterized by the membrane area, and noise due to inputs from other neurons in the network, i.e. synaptic noise. The cause of the destruction of the ISR effect as the gap junctional conductance increases is this latter noise source, as is evident in Fig 4. The top set of traces in each column, which are for high channel noise (i.e., small membrane area), are qualitatively the same. The behavior shown in the middle traces for low values of *g*_*e*_ corresponds to the trapping effect described above, in which the isolated neurons eventually get captured by the stable equilibrium and therefore cease firing. But as the gap junction conductance increases, synaptic noise soon becomes large enough to kick quiescent neurons back across the boundary and into the spiking state. We call this mechanism the **“kickout effect”**. As *g*_*e*_ increases, we see a restoration of intermittent firing states in the network, as is shown in the middle traces of the right column of Fig 4. Hence, for larger values of *g*_*e*_, the interaction among the neurons due to the network connectivity prevents the dip in the ISR curve from occurring, and the ISR effect is thus removed.

**Fig 5:**
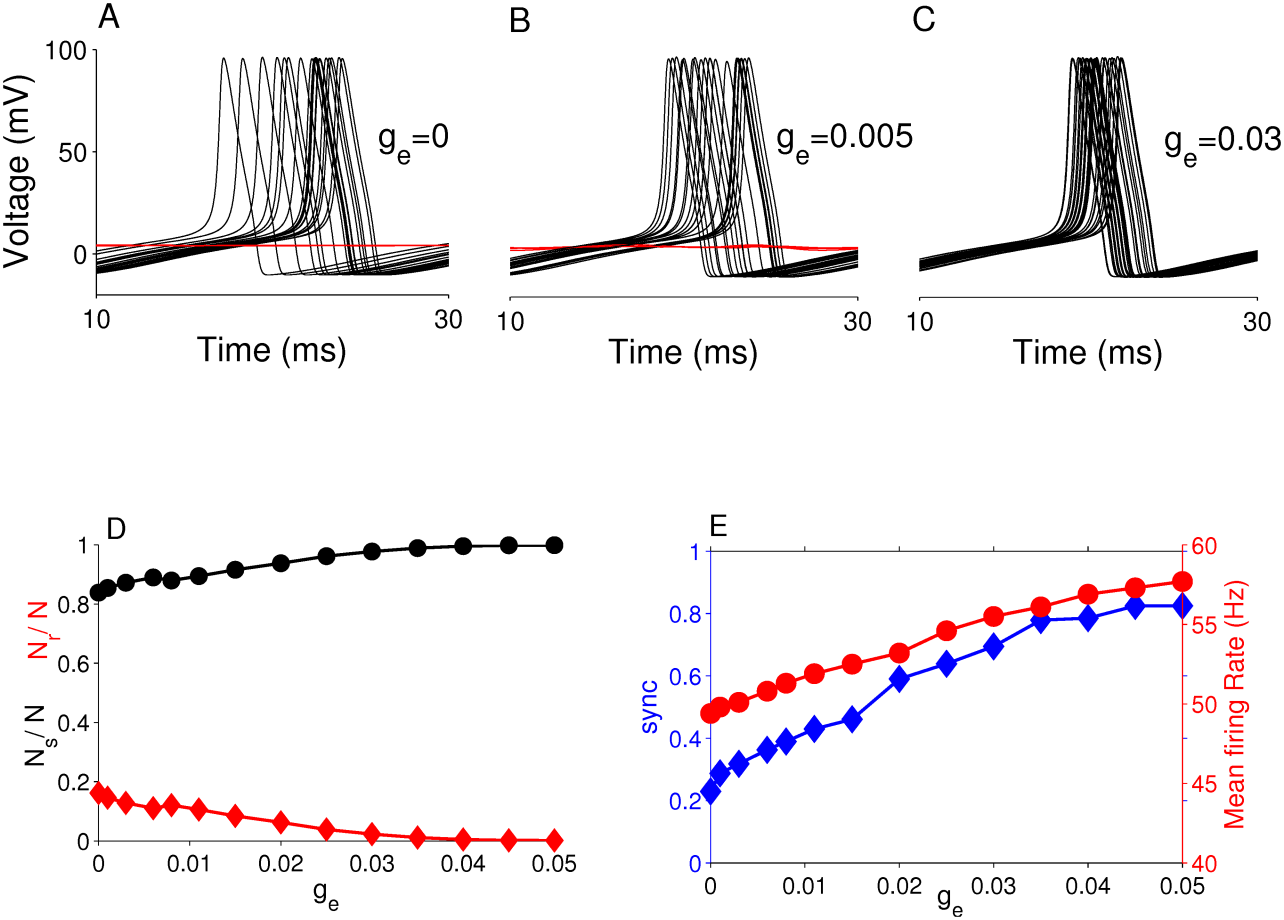
Factors shaping the ISR curves at low channel noise. Top panels: Voltage traces of 50 randomly-selected neurons from the networks corresponding to the bottom traces in Fig 4. (A) *g*_*e*_ = 0, (B) *g*_*e*_ = 0.005, (C) *g*_*e*_ = 0.03. Bottom panels: (D) The relative abundance of neurons in the resting (*N*_*r*_/*N*; red diamonds) and spiking (*N*_*s*_/*N*; black circles) states vs. *g*_*e*_. (E) The degree of synchronization in the network (blue diamonds, left vertical scale) and the global mean firing rate (red, right vertical scale) vs. *g*_*e*_. For all panels, *A* = 10^5^ *μm*^2^, 〈*k*〉 = 3, and *γ* = 3.

We now consider the network effects that lead to the increase in average firing rate seen at the extreme right sides of the ISR curves of Figs 1 and 2. This occurs with increasing *g*_*e*_, and is due to synchronization and recruitment of neurons into the spiking state. Panels A-C of Fig 5 show the voltage traces of several randomly-selected neurons from the networks corresponding to the bottom panels of Fig 4. We choose the low channel noise case (*A* = 10^5^ *μm*^2^) in order to evaluate more precisely the effect of network connectivity. Fig 5A corresponds to the network of isolated neurons, where *g*_*e*_ = 0. We see that because the neurons are initiated with random initial conditions, some neurons are in the resting state (red traces), and some are in the spiking state (black traces). Among the spiking neurons, there is a significant spread in the spike times.

In networks with increasing gap junction strength (Fig 5B and 5C), we see two effects. First, as *g*_*e*_ increases, more and more of the resting neurons get recruited into the spiking state via the network-driven kickout effect. Accordingly, the first neurons to be recruited are those with a high degree of connectivity, since they receive the strongest input from the network. For *g*_*e*_ = 0.03 (Fig 5C), all neurons are in the spiking state. Fig 5D shows the proportion of neurons in the whole network that are in the spiking (black circles) and resting (red diamonds) states versus the gap junction coupling strength, and the recruitment of resting neurons into the spiking state is clearly evident. Second, we observe that the degree of synchronization among the spiking neurons increases with *g*_*e*_. Fig 5E shows that synchronization (blue diamonds; left vertical scale) increases monotonically with *g*_*e*_. In addition, as more and more neurons enter the spiking state, we expect the mean firing rate of neurons across the network to increase. Accordingly, Fig 5E also shows that indeed, the mean firing rate of neurons in the network grows with *g*_*e*_ (red circles; right vertical scale), eventually reaching a value equal to that of an isolated spiking neuron.

### ISR in scale-free networks with chemical synapses

We also explored the emergence of ISR in scale-free networks of neurons with chemical synapses. We considered networks with only excitatory synapses, and networks with only inhibitory synapses. In both cases, ISR appears for some range of synaptic conductances, and we found that the phenomenon is more robust in networks with large inhibitory synaptic conductances than excitatory networks.

#### Networks with excitatory chemical synapses

**Fig 6:**
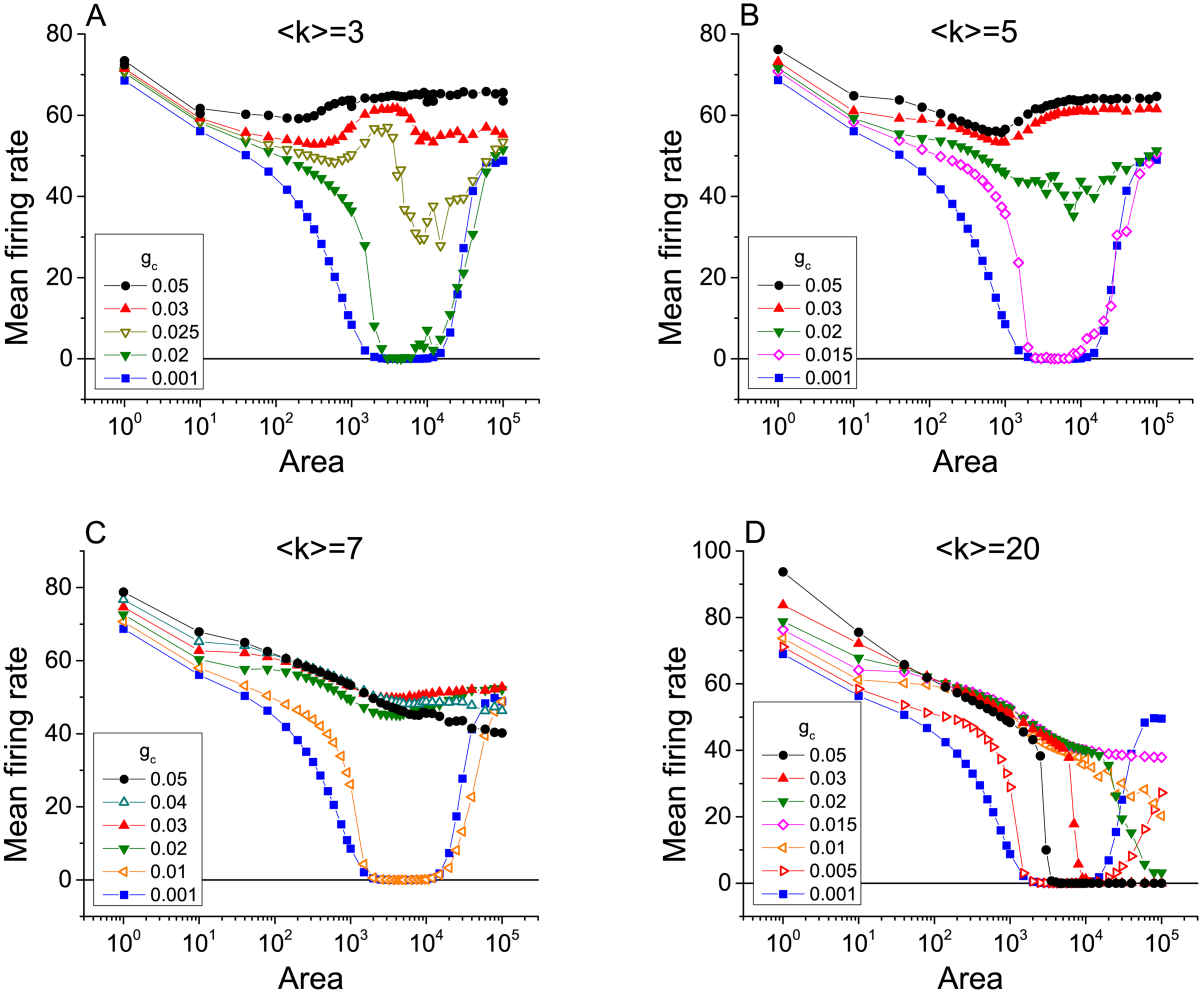
The emergence of ISR in scale-free networks of chemically-coupled neurons with excitatory connections. The mean firing rate as a function of cell membrane area for different values of the maximal synaptic conductance *g*_*c*_, with networks having degree distribution *p*(*k*) ∼ *k*^−*γ*^ and mean connectivity degree 〈*k*〉. Analysis has been performed in networks with fixed *γ* = 3, and four different values of 〈*k*〉, (A) 〈*k*〉 = 3, (B) 〈*k*〉 = 5, (C) 〈*k*〉 = 7, and (D) 〈*k*〉 = 20.

Fig 6 shows the mean firing rate plotted versus membrane area for a heterogeneous network with *γ* = 3 and 〈*k*〉 = 3, 5, 7, and 20, illustrating the emergence of ISR. The various curves in each plot represent different values of the maximal synaptic conductance *g*_*c*_. As in the case of electrical coupling, the ISR effect is most evident for the smallest values of connection strengths, for which the neurons in the network are essentially independent. ISR gradually goes away as the connectivity strength increases as in the case of electrical synapses due to the increasing role of network noise.

**Fig 7:**
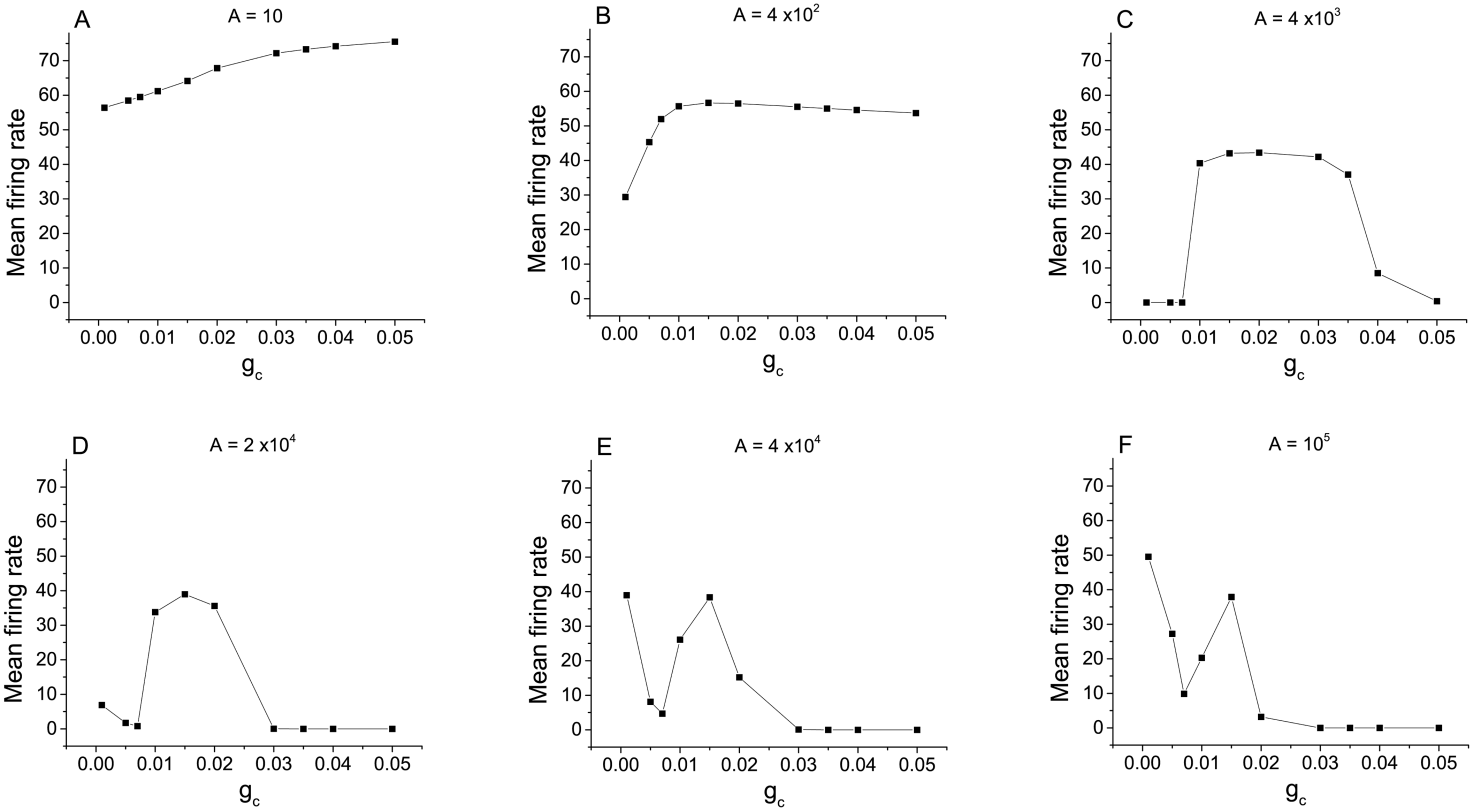
Determination of dynamical mechanism in a densely connected excitatory network. The mean firing rate versus coupling strength for chemically-coupled neurons with excitatory connections, with 〈*k*〉 = 20 and *γ* = 3, for different values of the membrane area as indicated in the panels. This is the same data as in Fig 6D, displayed differently.

Fig 6A and 6B are qualitatively similar to what we showed before for electrical synapses, and the mechanisms at work are essentially the same. However, for larger values of the average degree 〈*k*〉, new effects can be discerned. Note especially the differences between Fig 6A, for 〈*k*〉 = 3, and Fig 6D, for 〈*k*〉 = 20. These effects are more clearly apparent in Fig 7, which shows the data in Fig 6D as a sequence of graphs of the mean firing rate versus *g*_*c*_ for various values of the membrane area.

We begin by examining the left sides of all graphs in Fig 7, which correspond to low values of *g*_*c*_. Here we see the effects of the three dynamical mechanisms discussed above. First consider panel (F), which shows the case with the least channel noise (i.e, the largest membrane area). At *g*_*c*_ = 0, the mean firing rate is high due to the initial condition effect. As *g*_*c*_ increases, the mean firing rate initially decreases due to the trapping effect, eventually reaching a minimum value. As *g*_*c*_ continues to increase, the mean firing rate then increases due to the kickout effect driven by the increasing network synaptic noise. The subsequent collapse of the mean firing rate will be discussed below.

Now consider the left sides of the curves in reverse order (i.e., panels F, E, D, C, B, A), so that we see the consequences of increasing channel noise. The first effect is that the mean firing rate for low values of *g*_*c*_ decreases, indicating that the initial condition effect is being lost. The increasing channel noise is sufficient to cause trapping to occur in the absence of any significant network synaptic noise (i.e., for very low values of *g*_*c*_). Interestingly, however, the local minimum seen in panels F, E, D, and C remains at essentially the same value of *g*_*c*_. This suggests that the kickout effect that causes the mean firing rate to subsequently increase for increasing *g*_*c*_ is dominated by network synaptic noise. However, we see in panels B and A that when the channel noise is sufficiently large, it too can drive the kickout effect and cause the mean firing rate to increase. Thus, the left sides of the curves in Fig 7 can be understood in terms of the initial condition, trapping, and kickout effects.

In contrast, the behavior of the right sides of the curves for low channel noise (i.e., panels D, E, F of Fig 7) is caused by an additional dynamical mechanism. In this case, for large values of *g*_*c*_, strong network connectivity leads to spike synchronization among the neurons, and we observe that spiking in the network abruptly stops. This **“synchronization-induced termination”** has been previously reported in various contexts. Essentially, when the network is synchronized, coordinated synaptic inputs arrive at each neuron during the repolarization and refractory phase of the action potential. With sufficient synchronization, these inputs integrate into a large post-synaptic current that pushes the trajectory of each neuron into the basin of the resting equilibrium, and activity stops. Further analysis of similar behavior can be found in [43–45].

**Fig 8:**
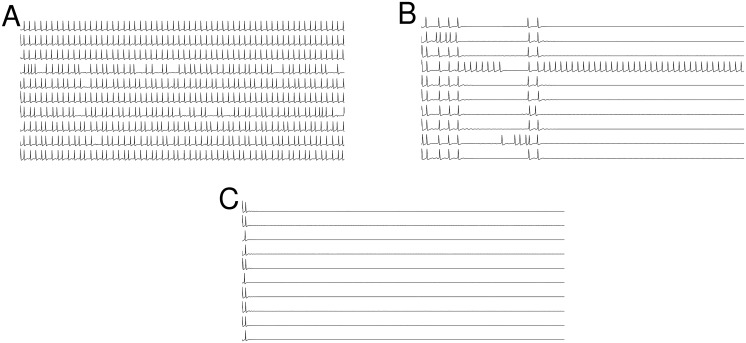
Enhanced synchronization leads to the termination of spiking activity in excitatory networks. Spike trains from ten randomly-selected neurons in a network with parameters as in Fig 7F, i.e., 〈*k*〉 = 20 and *γ* = 3, for *A* = 10^5^ and (A) *g*_*c*_ = 0.015, (B) *g*_*c*_ = 0.020, and (C) *g*_*c*_ = 0.025, depicting the emergence of the synchronization-induced termination effect as *g*_*c*_ increases.

To illustrate this, the three panels of Fig 8 show representative traces from ten randomly-selected neurons with parameters as in Fig 7F, for *g*_*c*_ = 0.015, 0.020, and 0.025. We see that the mean firing rate decreases dramatically as *g*_*c*_ increases. In Fig 8A, with *g*_*c*_ = 0.015, neuronal firing is consistent but with a bit of randomness. In Fig 8B, with *g*_*c*_ = 0.020, we see a few episodes in which many of the neurons temporarily stop firing. A close examination of the timing of the spikes before the onset of quiescent periods reveals that the last spikes are highly synchronized. After the final such event, all but one neuron has stopped firing. This neuron receives little or no synaptic input from the rest of the network, almost all of which is quiescent, and thus spikes at a rate close to the rate of spiking of an isolated neuron. Finally, in Fig 8C, with *g*_*c*_ = 0.025, a highly synchronous spiking event occurs right at the beginning of the traces, after which the neurons stop firing.

Returning now to Fig 7, we see the synchronization-termination effect on the right sides of the curves for low values of channel noise (i.e., large membrane areas; panels D, E, F). For the lowest channel noise (panel F), this effect is strongest, and we see zero firing rates persist for large *g*_*c*_ values as the channel noise increases (panels F, E, D). As the channel noise increases further, however, this noise gradually destroys synchronization. Lack of synchronization takes away the termination effect, and the large-amplitude noise ensures that many neurons spike persistently. Thus we see the right sides of the curves (i.e., high *g*_*c*_) go up (C, B, A) as the channel noise increases (i.e., membrane area decreases).

#### Networks with inhibitory chemical synapses

**Fig 9:**
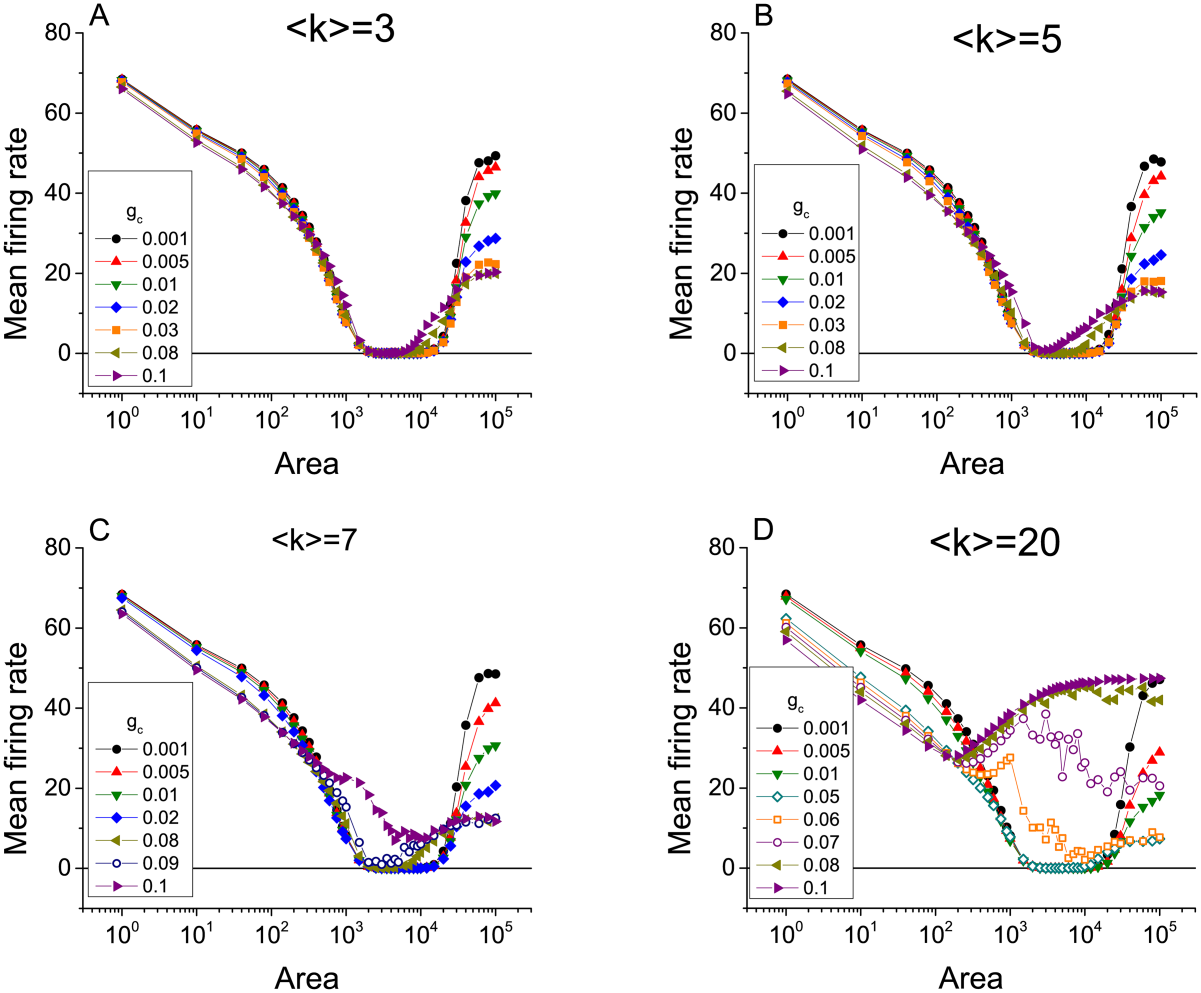
The emergence of ISR in scale-free networks of chemically-coupled neurons with inhibitory connections. The mean firing rate as a function of cell membrane area for different values of the maximal synaptic conductance *g*_*c*_, with inhibitory connected networks having degree distribution *p*(*k*) ∼ *k*^−*γ*^ and mean connectivity degree 〈*k*〉. Analysis has been performed in networks with fixed *γ* = 3 and 4 different values of 〈*k*〉, (A) 〈*k*〉 = 3, (B) 〈*k*〉 = 5, (C) 〈*k*〉 = 7, and (D) 〈*k*〉 = 20.

We also considered scale-free networks with inhibitory synapses. In this case, the ISR phenomenon also arises, but with some differences. Fig 9 illustrates plots similar to those shown above demonstrating the ISR effect for *γ* = 3, 〈*k*〉 = 3, 5, 7, and 20, and several values of *g*_*c*_. The most obvious new feature is that for 〈*k*〉 = 3, 5, and 7, the curves essentially lie on top of each other for *A* ≲ 10^4^. This indicates that for this parameter range, channel noise is dominant, and perhaps surprisingly, that synaptic noise is largely irrelevant except when the channel noise is very small (panels A, B, and C; *A* ≳ 10^4^). However, synaptic noise becomes important when the network is highly connected. This can be seen in panel D for 〈*k*〉 = 20, where the effect of *g*_*c*_ is evident for *A* ≳ 10^2^.

A careful examination of the right sides (i.e., low channel noise) in panels Fig 9A–9C reveals another difference between the inhibitory and excitatory cases for 〈*k*〉 = 3, 5, and 7. For networks with inhibitory synapses, increasing *g*_*c*_ results in a decrease in the mean firing rate, whereas in the analogous case with excitatory synapses, the opposite occurs. We previously observed that for excitatory synapses, increasing the network connection strength leads to a recruitment of neurons into the spiking state. Here we observe that for inhibitory synapses, increasing *g*_*c*_ leads to a recruitment of neurons into the resting state. This difference can be understood not only in terms of the opposite roles of excitatory and inhibitory synapses in the generation of action potentials, but also in terms of the bifurcation structure of the neurons and the mean values of the total synaptic current received by each neuron. For excitatory synapses, the mean synaptic current is positive. This causes the basin of the spiking state to increase in size as the unstable limit cycle decreases in amplitude. Thus, trapping becomes more difficult and the spiking state becomes more robust to synaptic current fluctuations. In the case of inhibitory synapses, the mean synaptic current is negative, and the effect is the opposite: the basin of the spiking state becomes smaller as the unstable limit cycle increases in amplitude, approaching the bifurcation that creates these limit cycles (saddle-node of periodic orbits). Trapping becomes more likely, and neurons fall into the resting state. Since an increase in *g*_*c*_ strengthens these effects in both cases, this explains the corresponding increase and decrease of the mean firing rates in the excitatory and inhibitory cases, respectively.

**Fig 10:**
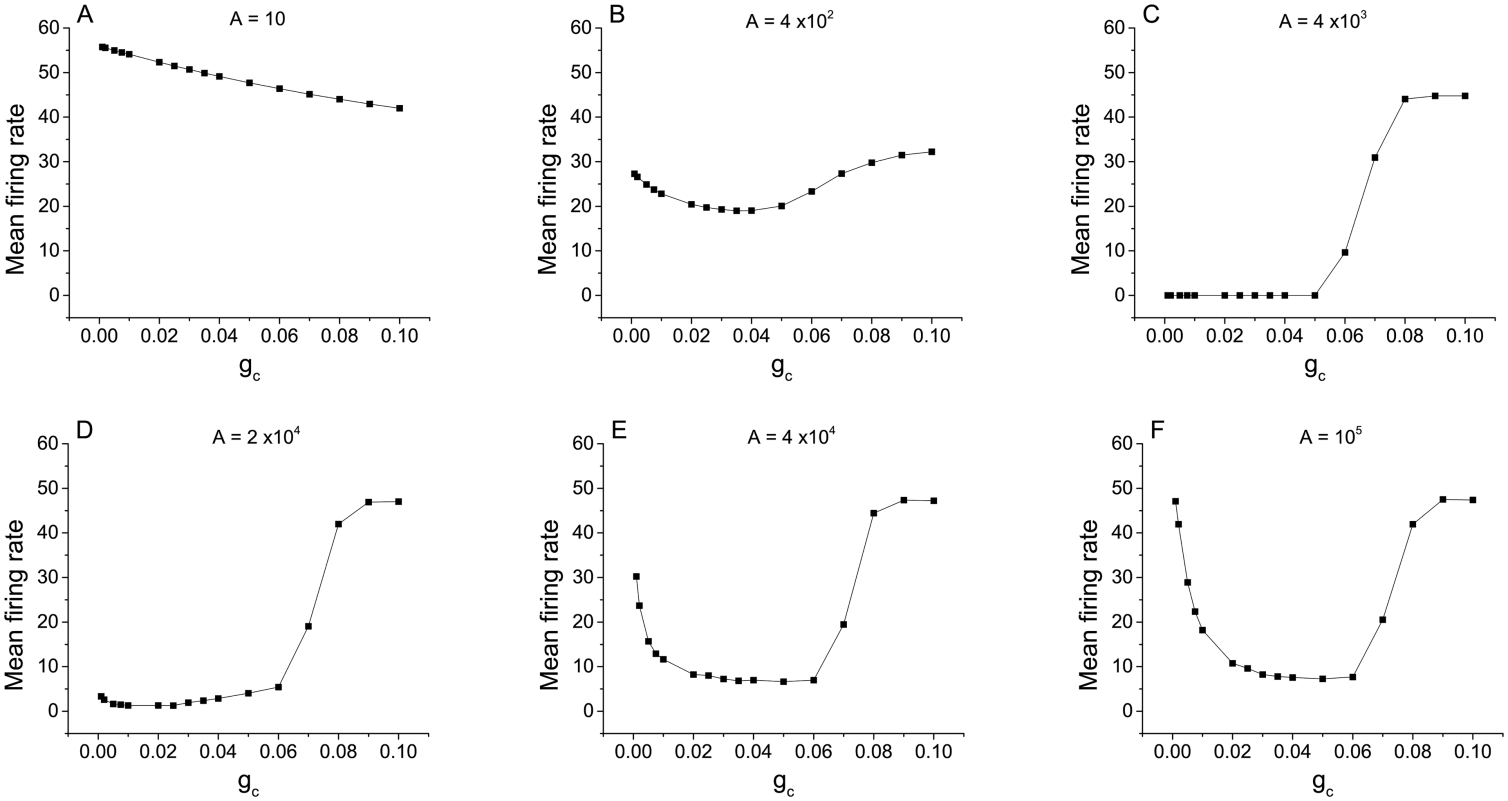
Determination of dynamical mechanism in a densely connected inhibitory network. Mean firing rate versus coupling strength for chemically-coupled neurons with inhibitory connections, with 〈*k*〉 = 20 and *γ* = 3, for different values of the membrane area as indicated in the panels. This is the same data as in Fig 9D, displayed differently.

As the mean connectivity degree 〈*k*〉 increases, different behavior emerges. For 〈*k*〉 = 7 and *A* = 10^5^ in the excitatory case (see Fig 6C), non-monotonic behavior appears as the synaptic strength is increased, but not in the inhibitory case (Fig 9C). For 〈*k*〉 = 20, the differences are significant. As before, we plot in Fig 10 the data of Fig 9D as graphs of mean firing rate versus *g*_*c*_ in order to tease apart the different mechanisms at play.

On the left sides of these curves, we observe the same scenario as before: For low network coupling and low channel noise, as in panel F, we see the initial condition effect, as well as the network-driven trapping effect that causes the mean firing rate to decrease as *g*_*c*_ increases. Trapping driven by channel noise is also observed for low *g*_*c*_ as membrane area increases (panels F, E, D, and C) until the channel noise induced kickout effect begins (panels B and A).

However, the right sides of these curves behave very differently from those in the case of excitatory networks. Recall that in the excitatory case, for high network connection strengths (*g*_*c*_) and relatively low channel noise, the mean firing rates fell to near zero due to the synchronization-induced termination effect described above. Here, in the inhibitory case, the mean firing rate approaches a high value as the network connection strength *g*_*c*_ increases. This is seen in all the panels, i.e., for all values of channel noise. An examination of the individual neurons in the networks at the highest value of *g*_*c*_ that we examined (*g*_*c*_ = 0.1) reveals that the neurons are highly desynchronized. In this state, spikes are mainly determined by synaptic current fluctuations causing random kickouts.

Finally, we note that in panel C, the mean firing rate is close to zero for *g*_*c*_ ≲ 0.06 due to the channel-noise driven trapping effect. The mean firing rate then rises for *g*_*c*_ ≳ 0.06 due to the network-noise driven kickout effect. This transition begins at approximately the same value of *g*_*c*_ in panels C-F, indicating that it is due essentially exclusively to network synaptic noise. In panels A and B, however, the channel noise is sufficiently high that this transition is lost.

### Influence of network size on ISR

**Fig 11:**
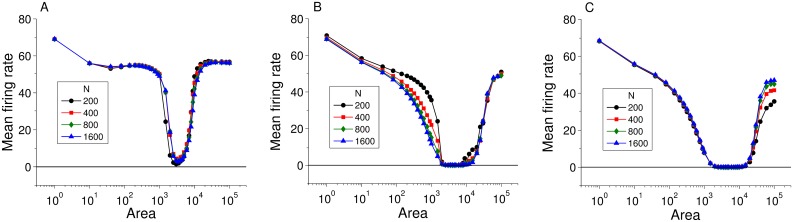
The influence of network size on the emergence of ISR in electrically and chemically coupled populations. Mean firing rate versus cell membrane area for different values of network size *N*, with 〈*k*〉 = 5. (A) Electrically coupled network with *γ* = 2.5 and *g*_*e*_ = 0.02. (B) Excitatory coupled network with *γ* = 3 and *g*_*c*_ = 0.015. (C) Inhibitory coupled network with *γ* = 3 and *g*_*c*_ = 0.01. The curves are almost identical for *N* = 200, 400, 800, and 1600 for all cases indicating that our results can be generalized for populations with larger sizes.

Finally, we investigated the effect of network size on the features of the ISR curve in the various configurations we considered above. Fig 11 shows ISR curves for heterogeneous scale-free networks with 〈*k*〉 = 5 and values of *γ* and the coupling strengths (*g*_*e*_ or *g*_*c*_) as indicated in the caption. The curves are for various values of *N*, the number of nodes in the network. We see no significant qualitative differences between these curves. Where there are differences, it is clear that as *N* increases, the curves asymptotically approach a shape that is in essence captured by the *N* = 200 case. So although there may be small quantitative differences in the results for networks of different sizes, we expect the same basic ISR profile with the same dynamical mechanisms at play to occur in the thermodynamic limit (*N* → ∞).

## Discussion

ISR is a recently-discovered phenomenon that has been investigated both theoretically and experimentally at the level of a single neuron [12–15, 18–20, 46]. In those studies, the average spiking rate of the neuron was calculated over many trials with the same neuron. In this work, we extended the concept of ISR to networks of many connected neurons by calculating the average spiking rate over the neuronal population instead. We found that indeed, ISR can emerge when formulated in this way. We showed that the important features of network-based ISR depend on the potentially complex interplay between the local ion channel noise of individual neurons and the effectively noisy post-synaptic inputs they receive from the rest of the network.

Essential to the occurrence of ISR is that this interplay between intrinsic and extrinsic sources of noise take place on a basic dynamical structure that underlies and governs the behavior of the neuron. Specifically, each neuron must exhibit a stable equilibrium that coexists with a limit cycle in such a way that the boundary between the basins of these attractors (which is mediated by an unstable periodic orbit) is very much closer to the limit cycle than it is to the equilibrium [19]. This dynamical picture is the basis for all types of ISR, and it leads to the curious behaviors we observed in this study.

In the classic Hodgkin-Huxley model neuron, this structure exists, in physical terms, for only a very small range of input currents. Thus one might be led to conclude that ISR would be difficult if not impossible to observe in real biological neurons. However, one must be careful when extrapolating from the point “space-clamped” HH neuron to real neurons with spatial extent and potentially complicated dendritic and axonal arborizations. And indeed, ISR behavior has recently been *experimentally* observed in individual Purkinje cells in slice preparations of rat brain [46]. This indicates that the necessary dynamical structure may not be hard to find after all.

When the intrinsic and extrinsic noise interacts with the attractor structure described above, various dynamical mechanisms can occur. We identified several such mechanisms that explain the shape of the ISR curves we calculated. With very low noise, we observed the initial condition effect, whereby a neuron approaches either the resting or the spiking state (depending on its initial conditions) and stays there. It does so because the noise is too small to perturb the neuron enough to change its state. With moderate noise, there is a higher likelihood that a neuron in the spiking state would be pushed into the resting state than the reverse. This is because of the close proximity of the basin boundary to the limit cycle, and the relatively large distance from that boundary to the resting equilibrium. Thus we observed the trapping effect, since neurons tend to get trapped in the resting state under these circumstances. Finally, with larger noise, neurons in the resting state can be kicked into the spiking state (and vice versa, of course). This is the kickout effect.

Based on these observations, we explain the main features of the ISR curve that occur as noise increases as follows. Beginning with no noise, a relatively high average spiking rate is observed due to the initial condition effect. This is followed under moderate noise by a drop in the average spiking rate due to the trapping effect. Finally, with large noise, a rise in the average spiking rate occurs due to the kickout effect.

We constructed our networks using scale-free topology, since this type of network includes two important ingredients observed in actual neural systems: scale invariance and small world characteristics [34, 47, 48]. However, this choice does not imply a loss of generality of our findings, since we observed that ISR is quite robust to moderate changes in network structure, e.g., varying the network heterogeneity and connection density. Thus, it is likely that ISR would appear in networks with other connection topologies as well. We also found that for networks larger than a minimum of approximately 200 neurons, the basic qualitative features of ISR behavior do not depend on the network size. Thus, one may expect ISR to appear in very large populations of neurons as in actual neuronal systems.

Our main result is that the interplay of channel noise (in individual neurons) and network noise (through network connections) affects the ISR phenomenon differently in the various scenarios we considered.

For networks of electrically coupled neurons, i.e. gap junctions, we found that it is possible to observe ISR as a function of channel noise when the network coupling is weak. In this case, channel noise is the dominant driver of the initial condition, trapping, and kickout effects in each neuron. However, as the network coupling between neurons increases, ISR gradually disappears as the dip in the average firing rate becomes less and less pronounced. The reason is that the increased noise amplitude due to the combination of the channel and network noises shifts the neurons into the regime of the kickout effect. This results in an increased average firing rate (see Fig 4). Of course the network noise input to each neuron depends on the network structure, and thus on the node degree distribution exponent *γ* and the mean connectivity 〈*k*〉. We found that ISR effects are less pronounced in more homogenous and more densely connected neuronal populations. This also happens with strong electrical connections.

For networks with purely excitatory chemical (i.e., synaptic) connections, we found the same effect: channel-noise driven ISR can be observed with low *g*_*c*_, and it tends to disappear as network noise increases with increasing *g*_*c*_. However, too much connectivity causes synchronization, and a new effect appears: synchronization-induced termination (SIT). The interesting mechanisms that we identified (i.e., the initial condition, trapping, and kickout effects) become irrelevant as the network activity is simply shut off. ISR then disappears, since SIT prevents the rising phase of the ISR curve. We found that sufficient synchronization to induce SIT occurs only with excitatory synapses and significant network connectivity, i.e., for large *g*_*c*_ and large values of 〈*k*〉.

For networks with purely inhibitory chemical (i.e., synaptic) connections, we found that channel-noise driven ISR is quite robust in the sense that the ISR curves are essentially independent of network effects (i.e., *g*_*c*_) when the connectivity (〈*k*〉) is moderate. Network effects tend to appear only when the channel noise is very small and/or the network connectivity is very large. When sufficiently large, these network effects tend to eliminate ISR because spiking events occur increasingly due to network noise -driven kickout events. Correspondingly, the network becomes highly unsynchronized.

The functional implications of ISR are not yet clear, nor have they been sufficiently explored in experiments since these observations are relatively recent. As mentioned above, ISR has been observed in a single real neuron via whole-cell patch clamp techniques. Our results suggest that ISR may be observable at the network level via measurement techniques that sample the activity of many neurons at once, such as local field potential measurements and possibly even EEG recordings. Since we found that ISR is most robust in networks dominated by inhibitory synaptic connections, perhaps the most promising approach to experimentally observing network ISR would be in well-controlled cell culture or brain slice experiments in which excitation is blocked, e.g., with tetrodotoxin.

It is widely assumed that information is processed with spikes. However, the silent periods characterizing ISR for certain conditions may play more than just an adverse role in neural systems. For instance, ISR may play a role in shortening the periods of anomalous working memory [49]. ISR may also play an important role in computational mechanisms that require on-off bursts of rhythmic spiking [50], and may provide a way for the behavior of a neuron (or a population thereof) that receives noisy input to be modulated without, or perhaps in conjunction with, more familiar chemical neuromodulators. It has also been proposed that Purkinje cells involved in cerebellar computation could use the ISR mechanism to switch among different operating regimes depending on input current fluctuations [46]. Furthermore, the low average firing rate of postsynaptic neurons in the ISR well may allow the filtering of irrelevant information, so that neurons can more selectively process information arriving through a different input channel. Thus, a better understanding of network ISR may be useful for understanding the complicated and interesting emergent dynamical behaviors that arise in real functioning neuronal systems.

In this work, we restricted consideration to networks with only one type of connectivity at a time. A natural extension would be to investigate network ISR in more realistic networks that feature several types of connectivity within the same population. For example, it is well-known that many real biological networks have mechanisms that maintain a balance of both excitatory and inhibitory synaptic activity, such as in the cortex of mammals [51, 52]. And of course, real networks of neurons coupled with both electrical and chemical synapses have also been extensively studied [53, 54]. This is important, as the dynamics of these types of populations have been shown to exhibit different characteristics in terms of synchronization and noise-driven behavior [55–58]. Furthermore, recent findings indicate that autaptic self-innervation in individual neurons, in addition to their presynaptic contacts from the network, play important functional roles in modulating population dynamics [59–61]. Thus, much work remains to be done to achieve a full understanding of how network structure shapes ISR behavior.
